# Mesenchymal Stromal Cells and Extracellular Vesicles: A Novel Therapeutic Paradigm for Mitochondrial Dysfunctions

**DOI:** 10.3390/ijms27041981

**Published:** 2026-02-19

**Authors:** Eman Salem Algariri, Fazlina Nordin, Min Hwei Ng, Izyan Mohd Idris, Norwahidah Abdul Karim, Gee Jun Tye, Wan Safwani Wan Kamarul Zaman

**Affiliations:** 1 Department of Tissue Engineering and Regenerative Medicine, Faculty of Medicine, Universiti Kebangsaan Malaysia, Cheras, Kuala Lumpur 56000, Malaysia; p131309@siswa.ukm.edu.my (E.S.A.); angela@hctm.ukm.edu.my (M.H.N.); 2 Institute for Medical Research (IMR), National Institutes of Health (NIH), Ministry of Health Malaysia, Shah Alam 40170, Selangor, Malaysia; izyan.idris@moh.gov.my; 3 Department of Biochemistry, Faculty of Medicine, Universiti Kebangsaan Malaysia, Cheras, Kuala Lumpur 56000, Malaysia; norwahidah@ukm.edu.my; 4 Institute for Research in Molecular Medicine (INFORMM), Universiti Sains Malaysia, Minden 11800, Penang, Malaysia; geejun@usm.my; 5 Malaysian Institute of Pharmaceuticals and Nutraceuticals, National Institutes of Biotechnology Malaysia, Gelugor 11700, Penang, Malaysia; 6 Department of Pharmaceutical Life Sciences, Faculty of Pharmacy, Universiti Malaya, Kuala Lumpur 50603, Malaysia; wansafwani@um.edu.my

**Keywords:** mitochondrial diseases, mitochondrial transfer, oxidative phosphorylation, MSC-base therapy, MSC-EVs, exosomes

## Abstract

Mitochondrial dysfunction is a central pathological feature of a wide range of inherited and acquired disorders and is characterized by impaired oxidative phosphorylation, disrupted cellular energy metabolism, and excessive oxidative stress. Although advances in molecular diagnostics have improved disease recognition, effective disease-modifying therapies remain limited, and clinical outcomes are often suboptimal, highlighting the need for novel therapeutic strategies. Mesenchymal stromal cells (MSCs) and their extracellular vesicles (MSC-EVs) have emerged as promising candidates for targeting mitochondrial dysfunction due to their regenerative, immunomodulatory, and metabolic regulatory properties. In this review, we provide a comprehensive overview of recent in vitro and in vivo studies investigating the capacity of MSCs and MSC-EVs to restore mitochondrial function by enhancing mitochondrial respiration, improving cellular bioenergetics, and reducing oxidative stress across diverse disease models. We further discuss the underlying mechanisms involved, including mitochondrial transfer, delivery of functional mitochondrial components, and modulation of the cellular microenvironment. Finally, we highlight the key advantages, translational potential, and remaining challenges associated with MSC- and MSC-EV-based therapies for mitochondrial dysfunction.

## 1. Introduction

Mitochondria are double-membrane organelles that function as central regulators of cellular metabolism, bioenergetics, and signaling. Structurally, they consist of an outer mitochondrial membrane that mediates metabolite exchange and protein import and an inner mitochondrial membrane folded into cristae, which contain the electron transport chain (ETC) complexes I–V and ATP synthase. The inner membrane encloses the mitochondrial matrix, which harbors mitochondrial DNA (mtDNA), ribosomes, and enzymes of the tricarboxylic acid (TCA) cycle and fatty acid β-oxidation [1]. Functionally, mitochondria generate ATP via oxidative phosphorylation (OXPHOS) by coupling electron transfer to proton gradient formation and ATP synthesis. Beyond energy production, mitochondria regulate reactive oxygen species (ROS) signaling, calcium homeostasis, apoptosis through cytochrome c release, innate immune responses, and cell fate decisions [2]. Mitochondrial dynamics—including fission, fusion, mitophagy, and biogenesis—are critical for maintaining mitochondrial quality control and adapting mitochondrial function to cellular metabolic demands [3].

**Figure 1:**
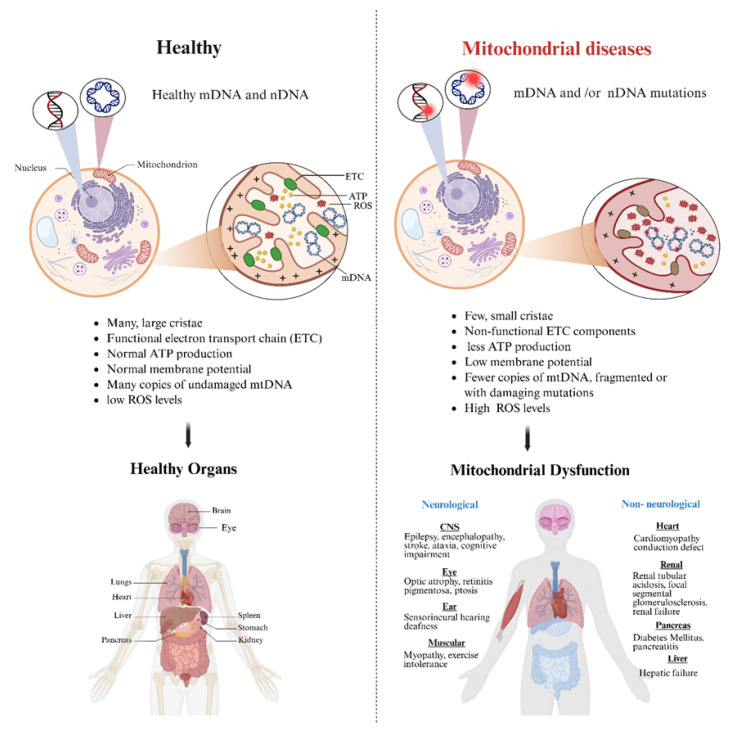
Healthy vs. mitochondrial dysfunction pathophysiology. In a healthy condition, mitochondria have many large cristae with normal membrane potential and functional ETC that result in supplying the cells with the required amount of ATP and keeping the ROS levels within normal healthy levels. In contrast, MD results from mtDNA and/or nDNA mutations that interrupt mitochondrial function by affecting the number and size of mitochondrial cristae, decreasing membrane potential, disturbing ETC component function, reducing ATP production, and increasing ROS levels, which leads to an increase in cellular oxidative stress and apoptosis and ends with cellular and organic dysfunction. Created in BioRender. Hasyahril, M. (2026). (https://BioRender.com/4ofqv30). (accessed on 18 November 2025).

Consequently, defects in mitochondrial structure, bioenergetic pathways, signaling functions, or dynamic processes can lead to mitochondrial dysfunctions (MDs), ultimately disrupting cellular homeostasis and contributing to the pathogenesis of a wide spectrum of metabolic, neurodegenerative, cardiovascular, and genetic disorders. Mitochondrial dysfunctions are broadly classified into two categories: primary mitochondrial dysfunctions/diseases (PMDs), which are groups of genetic disorders caused by inherited mutations in nuclear or mitochondrial genes that encode structural OXPHOS proteins or proteins required for OXPHOS function, and secondary mitochondrial dysfunctions (SMDs), which arise as a downstream consequence of non-mitochondrial genetic defects, environmental factors, aging, and chronic or inflammatory diseases [4,5]. The hallmarks of mitochondrial dysfunctions are energy deficits, increased oxidative stress, loss of mitochondrial membrane potential, and activation of apoptotic pathways (Figure 1). PMDs are characterized by complex genetics, multisystemic involvement, and significant diagnostic challenges; a single syndrome may result from mutations in multiple genes, while a single variant may lead to widely variable clinical presentations [6]. PMDs affect approximately 1 in 5000 adults worldwide, with asymptomatic carriers reaching nearly 1 in 250 in the general population [7,8]. Due to their multisystemic impact, progressive course, and absence of curative therapies, MDs impose a substantial disease burden and highlight the urgent need for strategies capable of restoring mitochondrial function and improving organ performance.

Mesenchymal stromal cells (MSCs) have emerged as promising candidates in regenerative medicine due to their immunomodulatory properties, paracrine activity, and ability to transfer healthy organelles and bioactive molecules to damaged cells [9]. Notably, accumulating evidence indicates that many of the therapeutic benefits of MSCs are mediated through their secretome, particularly extracellular vesicles (EVs), rather than direct cell engraftment [10]. MSC-derived EVs (MSC-EVs), including exosomes, encapsulate a diverse cargo of proteins, lipids, mRNAs, microRNAs, and, in some cases, mitochondrial components or whole mitochondria, enabling them to modulate inflammation, oxidative stress, apoptosis, and cellular metabolism [11]. Both MSCs and MSC-EVs are emerging as attractive therapeutic platforms for mitochondrial restoration due to their ability to transfer bioactive molecules and mitochondrial components to injured cells. While MSCs can directly enhance tissue repair and mitochondrial function, growing evidence suggests that MSC-EVs mediate many of these effects and represent a safer, cell-free alternative with significant potential for rescuing mitochondrial dysfunction [11,12].

Therefore, this review aims to evaluate emerging therapeutic strategies based on mesenchymal stromal cells (MSCs) and their extracellular vesicles (MSC-EVs) for the treatment of mitochondrial dysfunctions. This review synthesizes current experimental and preclinical evidence regarding their capacity to restore mitochondrial function, enhance cellular bioenergetics, and attenuate oxidative stress and inflammation. Furthermore, it examines the underlying molecular mechanisms, discusses translational potential, and highlights key limitations that must be addressed for clinical application.

## 2. Genetics of Mitochondrial Dysfunctions

Mitochondrial DNA (mtDNA) is a small, circular, double-stranded genome located inside mitochondria, the organelles responsible for producing cellular energy through oxidative phosphorylation (OXPHOS). Unlike nuclear DNA, mtDNA is inherited almost exclusively from the mother and encodes 37 genes, including 13 proteins essential for the mitochondrial respiratory chain, 22 transfer RNAs, and 2 ribosomal RNAs [13,14]. The nuclear genome encodes approximately 250–300 proteins that localize to the mitochondria. These proteins are synthesized in the cytoplasm and transported into the organelle through an intricate protein import system. In total, an estimated 1500 nuclear genes are involved in mitochondrial processes, including functions beyond the electron transport chain (ETC) [15,16,17]. mtDNA has a high mutation rate due to limited DNA repair mechanisms and its proximity to reactive oxygen species, with the mitochondrial genome exhibiting a mutation rate approximately 100–1000 times higher than that of the nuclear genome, making it a major contributor to mitochondrial dysfunction [18,19]. Given the dual genetic control of mitochondrial function, pathogenic variants in mtDNA and/or nDNA can impair energy production and cause a wide range of multisystemic disorders affecting high-energy-demand tissues, such as the brain, heart, and skeletal muscle [4,5,20].

Primary mitochondrial diseases are caused by genetic mutations involved in mtDNA or nDNA genes that encode OXPHOS structure and function proteins [4]. Pathogenic variants can be inherited through autosomal recessive, autosomal dominant, maternal, or X-linked patterns [15]. PMD manifests in a wide range of clinical syndromes. Among the most frequently reported are mitochondrial myopathy, encephalopathy, lactic acidosis, stroke-like episodes (MELAS), Leber hereditary optic neuropathy (LHON), Kearns–Sayre syndrome (KSS syndrome), progressive extraocular muscle paralysis (PEO), and Leigh syndrome [7]. PMDs have a complex genetic basis, in which specific syndromes may arise from diverse genetic etiologies. For example, Leigh syndrome can result from a variety of mtDNA and nDNA mutations, including pathogenic variants in MT-ND1, MT-ND3, MT-ND4, and MT-ND6; nuclear defects such as NDUFS1; or a combination of both [4]. Conversely, a single mtDNA mutation may lead to multiple clinical presentations. A well-recognized example is the m.3243A>G variant in MT-TL1, which can manifest as PEO, MELAS, or maternally inherited diabetes and deafness (MIDD) [5,18].

In addition, the severity of mitochondrial diseases is strongly influenced by the degree of heteroplasmy and the tissues affected. Mutation m.8993 T>G or m.8993 T>C in the *MT-ATP6* gene is implicated in the pathogenesis of neuropathy, ataxia, and retinitis pigmentosa (NARP). These mutations are also associated with Leigh syndrome, with the specific phenotype determined by the level of heteroplasmy. Levels exceeding 85% are predominantly linked to childhood-onset Leigh syndrome, whereas a level of 60–70% typically results in adult-onset NARP. Both diseases may occur with heteroplasmy levels between 70 and 85% [21,22]. Similarly, the m.13094T>C variant shows that high mutation loads are associated with severe Leigh syndrome, while lower heteroplasmy produces a broader spectrum of neurological symptoms [23].

Secondary mitochondrial dysfunction (SMD) refers to mitochondrial impairment that occurs as a downstream consequence of non-mitochondrial genetic defects, systemic diseases, environmental stressors, or aging, rather than primary mutations in oxidative phosphorylation (OXPHOS) genes. In SMD, the primary pathological insult indirectly disrupts mitochondrial bioenergetics, redox homeostasis, and quality control pathways, thereby contributing to disease progression and tissue degeneration [24,25,26]. SMD is frequently implicated in multifactorial disorders such as diabetes, cardiovascular disease, cancer, kidney disease, and neurodegenerative conditions [24,27,28,29,30]. Genetic alterations in SMD frequently involve nuclear genes that regulate mitochondrial dynamics, quality control, and metabolic pathways rather than core respiratory chain subunits. Mutations in genes controlling mitochondrial fusion and fission, such as MFN2, OPA1, and DNM1L, result in fragmented or dysfunctional mitochondrial networks that compromise ATP production and intracellular mitochondrial distribution [31,32]. Similarly, defects in mitophagy-related genes, including PINK1 and PRKN, lead to impaired clearance of damaged mitochondria, promoting oxidative stress and cellular degeneration, particularly in neurodegenerative disorders [33,34].

Alterations in genes involved in mitochondrial iron–sulfur cluster biogenesis, such as FXN, further contribute to secondary respiratory chain dysfunction and oxidative stress, as observed in Friedreich’s ataxia [35]. Moreover, mutations in genes responsible for mtDNA maintenance, including POLG, TWNK, and MPV17, can cause mtDNA depletion or deletions, resulting in secondary OXPHOS defects [36]. At the signaling level, SMD is strongly influenced by dysregulated metabolic and stress response pathways. The PGC-1α signaling axis, which controls mitochondrial biogenesis and oxidative metabolism, is frequently suppressed in metabolic and neurodegenerative diseases, leading to reduced mitochondrial mass and respiratory capacity [37,38]. Energy-sensing pathways such as AMPK and nutrient-sensing pathways such as mTOR modulate mitochondrial biogenesis, autophagy, and mitophagy, and their chronic dysregulation contributes to mitochondrial dysfunction in diabetes, aging, and cancer [39,40]. Inflammatory signaling pathways, including NF-κB and the NLRP3 inflammasome, are activated by mitochondrial ROS and mtDNA release, further exacerbating mitochondrial damage and chronic inflammation [41]. Table 1 summarizes the key genetic basis, pathophysiological mechanisms, and key clinical features of selected examples of primary and secondary mitochondrial dysfunctions.

## 3. Diagnosis of Mitochondrial Dysfunctions

Mitochondrial diseases are genetically complex and can present with a wide spectrum of clinical manifestations, making diagnosis challenging. Their presentation may be neurological or non-neurological, with onset ranging from acute or subacute to slowly progressive. Neurological features include seizures, ataxia, stroke-like episodes, encephalopathy, myopathy, muscle weakness, optic atrophy, retinitis pigmentosa, and auditory neuropathy. Non-neurological manifestations reflect multisystem involvement and may include hypertrophic or dilated cardiomyopathy, Fanconi syndrome, diabetes mellitus, and premature ovarian failure [52,53,54,55]. Childhood mitochondrial diseases are often associated with diverse clinical presentations, particularly in children under two years of age [56]. Common features include fatigue, vomiting, failure to thrive, hypotonia, encephalopathy, and seizures [57]. Some mitochondrial disorders follow a characteristic clinical course, such as Pearson syndrome (PS), which initially presents with severe hypoproliferative anemia in early infancy and later progresses to multi-organ dysfunction, including lactic acidosis, pancreatic insufficiency, renal tubulopathy, failure to thrive, muscle hypotonia, and endocrine abnormalities [21,22]. During disease progression, anemia may spontaneously resolve in some PS patients, whereas others may evolve into Leigh syndrome or Kearns–Sayre syndrome [22]. Because mitochondrial diseases can mimic many common conditions due to their multisystem involvement, they are frequently considered in the differential diagnosis of diverse disorders. Therefore, confirmatory analyses—including biochemical testing and molecular genetic evaluation—are essential for achieving an accurate diagnosis.

A variety of biochemical screening tests can support the diagnosis of mitochondrial diseases. These include complete blood counts, urine organic and amino acid analyses, hormone screening, hemoglobin A1C, comprehensive metabolic panels, measurements of blood lactate and pyruvate, and assessments of creatine kinase, ammonia, carnitine, acylcarnitine, and lipoprotein profiles [53]. Molecular genetic testing is a critical component of the diagnostic process, as it enables identification of the underlying molecular etiology of mitochondrial dysfunction and guides therapeutic decision-making. The first-line molecular diagnostic test for mitochondrial disease typically involves next-generation sequencing (NGS) of mitochondrial DNA (mtDNA) to detect point mutations, deletions, and heteroplasmic variants associated with primary mitochondrial diseases [58,59,60]. If mtDNA sequencing does not identify a causative mutation, whole-exome sequencing (WES) or whole-genome sequencing (WGS) can be used to detect pathogenic variants in nuclear genes encoding mitochondrial proteins [58,59]. In addition to genetic analysis, tissue-based investigations—such as biochemical assays of respiratory chain enzyme activity and histopathological evaluation of skeletal muscle or skin biopsies—can provide supportive or confirmatory evidence of mitochondrial dysfunction, particularly when molecular findings are inconclusive [58,60].

## 4. Treatment and Prognosis of Mitochondrial Dysfunctions

The treatment of mitochondrial diseases remains challenging due to their genetic heterogeneity, multisystem involvement, and the limited availability of disease-modifying therapies. Currently, most therapeutic approaches are supportive and aim to improve mitochondrial bioenergetics, reduce oxidative stress, and manage organ-specific complications. Conventional management strategies include metabolic supplementation and symptomatic treatment. Nutritional and pharmacological agents such as coenzyme Q10 (CoQ10), riboflavin, L-carnitine, thiamine, and antioxidant vitamins are commonly used to enhance electron transport chain (ETC) activity, improve fatty acid oxidation, and mitigate oxidative stress [5,61,62,63,64,65,66,67,68,69,70]. In clinical practice, these agents are often administered as combinations of three to six compounds, commonly referred to as “cocktail therapy.” Although this approach may provide modest symptomatic relief, its overall effectiveness remains limited, and the composition of such regimens varies considerably without standardized protocols.

Condition-targeted interventions include idebenone for Leber hereditary optic neuropathy (LHON) and L-carnitine and arginine/citrulline supplementation for MELAS to reduce stroke-like episodes [65,66]. Symptomatic therapies, including antiepileptic drugs, cardiac pacing, and physiotherapy, are essential for managing neurological, cardiac, and muscular manifestations [61,65]. While the clinical use of these strategies is guided by current insights into mitochondrial disease pathophysiology, the supporting evidence for their therapeutic efficacy remains limited. Table 2 summarizes the conventional and current therapeutic strategies for mitochondrial diseases. 

Despite these supportive strategies, the overall prognosis of mitochondrial diseases remains poor, particularly in pediatric cases [76,77]. A systematic review of the natural history of these disorders reported that most cases begin in childhood, with 59% presenting before 18 months of age and 81% before 18 years. Mortality is high, with 13% of patients dying before 1 year, 57% before 5 years, and 74% before 10 years [76]. A retrospective observational study of neonatal mitochondrial diseases found neonatal onset in 28.7% of cases, with an overall mortality rate of 44.8%. Mortality was especially high in patients with cardiomyopathy (60.5%) compared to those with Leigh syndrome (23.1%). The median survival time was only 1.86 years, with a one-year survival rate of 51.8% [77]. These findings underscore the urgent need for effective therapies that move beyond symptomatic management to directly target the underlying defects to restore mitochondrial function. Among the emerging strategies, mesenchymal stromal cell (MSC)-based and MSC-derived extracellular vesicle (MSC-EV)-based therapies have gained significant attention for their potential to achieve such restorative effects, as discussed in the following sections.

## 5. Therapeutic Potential of Mesenchymal Stromal Cells and Their Extracellular Vesicles in Mitochondrial Dysfunctions

In recent decades, stromal cell therapy has emerged as a promising therapeutic approach. Mesenchymal stromal cells (MSCs) derived from various sources possess several advantageous properties, including low immunogenicity, potent paracrine signaling, antioxidant effects, and the unique ability to migrate to sites of injury and transfer mitochondria [9]. A growing body of evidence from in vitro, in vivo, and clinical studies has consistently demonstrated the therapeutic potential of MSCs across a wide range of degenerative and metabolic disorders, such as diabetes mellitus [78], osteoarthritis [79], wound healing [11], degenerative disc disease [80], and retinal degenerative disease [81]. A recent clinical trial on type-1 diabetes reported that MSC transplantation was safe, improved HbA1c and C-peptide levels, and shifted pro-inflammatory cytokines toward an anti-inflammatory profile [78]. Similarly, a systematic review evaluating MSC therapy for osteoarthritis (OA) concluded that MSCs are safe and effective in reducing pain and improving knee function in patients with knee OA [82]. Furthermore, MSC-derived exosomes—specialized extracellular vesicles—have shown superior therapeutic efficacy compared with MSCs themselves in models of cardiac ischemia–reperfusion injury and diabetic wound regeneration [11,12].

Taken together, these findings highlight the broad therapeutic potential of MSCs and their extracellular vesicles (MSC-EVs) across diverse disease models. Although current evidence underscores their regenerative and protective roles, the application of MSC-based therapies in the context of mitochondrial diseases remains relatively limited. Therefore, the following sections of this review summarize the available in vitro and in vivo studies investigating the therapeutic effects of MSCs and MSC-EVs in mitochondrial diseases.

### 5.1. Therapeutic Potential of Mesenchymal Stromal Cells in Mitochondrial Dysfunctions

Mesenchymal stromal cells have gained increasing attention for their ability to repair cellular damage and restore mitochondrial function through mechanisms such as paracrine signaling, antioxidant activity, and mitochondrial transfer. Although research on MSC-based therapies for mitochondrial diseases remains limited and is still emerging, it demonstrates promising therapeutic potential. Liu et al. investigated the effects of bone marrow-derived mesenchymal stromal cells (BM-MSCs) and highly purified mesenchymal stromal cells (RECs) in iPSC-derived neurons from patients with MELAS. Both direct and indirect co-culture with MELAS neurons significantly restored mitochondrial function, primarily through mitochondrial transfer from MSCs [83]. This intervention improved mitochondrial membrane potential, ATP production, ROS regulation, intracellular calcium homeostasis, and oxygen consumption rate. The transfer of intact mitochondria and/or mitochondrial components was mediated by tunneling nanotubes (TNTs), connexin-43-mediated gap junction channels (Cx43-GJCs), and extracellular vehicles (EVs) [83].

In another study, co-culture of BM-MSCs with complex I-deficient fibroblasts revealed a significant improvement of mitochondrial respiration, reduced cellular ROS levels, and upregulation of antioxidant enzymes such as SOD2 (superoxide dismutase 2, mitochondrial) and HO-1 (heme oxygenase 1) [84]. Notably, repeated co-culture with MSCs at two sequential time points further enhanced the therapeutic effects, with a more sustained reduction in ROS levels observed after direct co-culture compared to treatment using MSC-conditioned medium alone. Furthermore, co-culture of iPSC-derived neural progenitor cells (NPCs) from an LHON patient with MSCs led to marked mitochondrial genomic improvements, including an increased ratio of wild-type to mutant mitochondrial DNA (mtDNA), elevated levels of the wild-type mtND4 (m.11778A allele), and reduced expression of the mutant mtND4 (m.11778A>G allele) [85]. These genomic shifts were accompanied by a significant enhancement in mitochondrial metabolic function in LHON-derived neurons.

Mitochondrial dysfunction is a central contributor to the pathogenesis of diabetic nephropathy [51], and repeated administration of MSCs has been shown to ameliorate tubular epithelial cell injury and delay disease progression by enhancing mitochondrial function and modulating inflammatory responses [86,87]. Extending these findings to neurological disorders, cerebral ischemia/reperfusion (I/R) injury—a condition marked by severe neuroinflammation and mitochondrial dysfunction [88]—was shown to benefit from treatment with ischemic-hypoxic preconditioned olfactory mucosa-derived MSCs. This treatment exerted significant neuroprotective effects in vitro and in vivo, including reduced infarction, improved motor outcomes, and preservation of mitochondrial integrity [89]. Similar therapeutic benefits have been reported in retinal degenerative disease, where mitochondrial dysfunction and Müller cell gliosis are major pathological features [90]. Treatment of Müller cells with BM-MSCs enhanced mitochondrial function, reduced oxidative stress and gliosis, and partially preserved visual function in degenerative rat retinas [91]. These effects may be attributed to increased mitochondrial DNA (mtDNA) content and the promotion of mitochondrial fusion in damaged Müller cells. Further details of these studies are presented in Table 3.

### 5.2. Therapeutic Potential of MSC-Derived Extracellular Vesicles in Mitochondrial Dysfunctions

Although many studies have highlighted the regenerative potential of MSC-derived extracellular vesicles, recent investigations have increasingly emphasized their role in alleviating mitochondrial dysfunction, particularly in secondary mitochondrial disorders. Intervertebral disc degeneration (IVDD), whether age-related or induced by genetic and mechanical factors, is strongly associated with mitochondrial impairment, which promotes excessive ROS production, leading to extracellular matrix degradation, loss of nucleus pulposus cells (NPCs), and enhanced inflammatory signaling [92,93,94,95]. In vitro studies demonstrated that umbilical cord MSC-exosomes (UC-MSC-exos) enhance NPC viability, reduce intracellular and mitochondrial ROS, and restore mitochondrial membrane potential, while in vivo evidence shows their ability to slow IVDD progression in rat models [94,95]. Similarly, mitochondrial dysfunction plays a central role in the pathogenesis of kidney disease [96], in which MSC-EVs have been shown to attenuate mitochondrial injury by inhibiting fission, enhancing antioxidant defenses, and promoting ATP production [97]. Notably, hypoxia-preconditioned EVs (Hypo-EVs) exhibit superior therapeutic efficacy in restoring renal function and reducing fibrosis [98]. Further studies demonstrating the mitochondrial-enhancing effects of MSC-EVs are summarized in Table 3.

Therapeutic efficacy evidence of MSC-EVs in genetically defined PMDs is currently very limited. A recent landmark study demonstrated that mitochondria-enriched MSC-EVs (EV-Mito) restored mitochondrial membrane potential, ATP production, and oxidative phosphorylation while reducing mitochondrial reactive oxygen species in Leber hereditary optic neuropathy (LHON) mtDNA-mutant cells and animal models [99]. Collectively, these findings highlight a critical research gap in the field, underscoring the need for further investigation of MSC-EV-based therapies in additional genetically defined primary mitochondrial diseases. While promising therapeutic efficacy has been demonstrated in LHON models, it remains unclear whether MSC-EV-mediated mitochondrial transfer and bioenergetic rescue can be generalized to other PMDs, such as Leigh syndrome, MELAS, or mitochondrial DNA depletion syndromes. Therefore, systematic preclinical studies in diverse PMD models are required to validate the therapeutic potential, disease specificity, and mechanistic efficacy of MSC-EVs as a universal mitochondrial-targeted regenerative strategy.

**Table 3:** MSC and MSC-EV therapeutic potential for mitochondrial dysfunctions: in vitro and in vivo studies.

Source of MSCs/MSC-EVs	Targeted Disease	Targeted Cells/Animal Model	Dosage/Route of Administration	Therapeutic Effects	Mechanism	Ref.
Human BM-MSCs & highly purified MSCs (RECs)	MELAS syndrome	iPSC-derived MELAS neurons	Not specified	- Restore mitochondrial membrane potential - Improve ATP production - Reduce ROS levels - Restore intracellular calcium storage - Restore oxygen consumption rate	- Mitochondria donation	[83]
Human MSCs	Leber’s hereditary optic neuropathy (LHON)	LHON iPSC-derived NPCs	1:1 ratio MSC: NPCs	- Increase mitochondrial respiration and ATP production	- Mitochondria donation - Increase the ratio of normal mtDNA to mutant mtDNA	[85]
Human BM-MSCs	Complex I deficiency	Human fibroblast with MT-ND3 & MT-ND6	Not specified	- Improve mitochondrial respiration - Reduce ROS levels	- Mitochondria donation - Upregulation of cellular antioxidant, SOD2 and HO-1	[84]
Human UC-MSCs	Diabetic nephropathy	Murine macrophage cell line (RAW264.7 cells)	1:2 ratio MSCs: RAW264.7 cells	- Anti-inflammatory effect - Improve mitochondrial function - Reverse albuminuria and prevent the progression of diabetic nephropathy	- Mitochondria donation - Increase *Arg1* expression and suppressed M1 polarization in macrophages - Reverse cytokine-mediated mitochondrial dysfunction	[86]
		8-week-old male CD1 mouse model of diabetic nephropathy	Mice were injected intravenously with 5.0 × 10^5^ UC-MSCs thrice every 4 weeks			
Human and murine BM-MSCs	Diabetic nephropathy	Human podocytes	1:1 ratio MSCs: podocytes	- Improve mitochondrial function - Improve renal function	- Mitochondria donation - Reduce mitochondrial damage - Reduce apoptosis and inflammation - Increase nephrin gene expression	[87]
		8-week-old male C57BL6 mouse model of diabetic nephropathy	Mice were injected via tail vein, with BM-MSCs (1.0 × 10^4^ cells/g body weight) once a week for 6 consecutive weeks			
Human OM- MSCs	Cerebral ischemia/reperfusion injury	Neuron (SH-SY5Y) cells	Not specified	- Improve neuron mitochondrial function (increase MMP and decrease ROS) - Inhibit apoptosis and pyroptosis of neurons - Reduce damaged areas of the infarct cortices and improve rat motor function	- Increase GRP78 and Bcl-2 proteins - Decrease NLRP3 inflammasome and pyroptosis-associated proteins, ASC, caspase1, caspase8 and GSDMD - Decrease BAX, IL-1β and IL-18	[89]
		Adult Sprague–Dawley rat model of Cerebral ischemia/reperfusion injury	IhOM-MSCs (1 × 10^6^) were injected into the rat tail vein			
Mouse Ad-MSC-EVs	Leber’s hereditary optic neuropathy (LHON)	LHON model cells (GM10742 cells)	6 μg EV-Mito protein/well	- Restore mitochondrial functions (MMP, ATP production, mitochondrial ROS levels, and mPTP opening) - Enhance the proliferative capacity of LHON model cells - Enhance visual recovery in LHON mice	- Mitochondria donation - Increase expression of ND4 and COX IV proteins	[99]
		3-month-old mutant mtND4R340H mtTg LHON male mice	Mice received an intravitreal injection of 1 μg EV-Mito protein/eye, 2 times administrations			
Human UC-MSC-EV	Cardiac hypertrophy	Neonatal rat cardiac myocytes (from 1- to 3-day-old Sprague–Dawley rats) as model of cardiac hypertrophy	- 100 ug/mL Nor-EVs or Hypo-EVs	- Reduce the cardiomyocyte size - Improvement of mitochondrial function - Attenuate heart size, ventricular wall thickening and cardiomyocyte cross-sectional areas	- Transfer DJ-1 protein to cardiomyocytes - Decrease mRNA expression of hypertrophic indicators (BNP, ANP, and β-MHC) - Enhance expression of antioxidant-related proteins, such as NRF2, HO-1, SOD2 and GPX4, while downregulate expression of NOX4 - Upregulate the expression of p-AMPKα/AMPKα and PGC-1α - Upregulate the expression of ATRAP, which inhibits the activation of p38 and ERK1/2 signaling pathways	[100]
		Male 8-week-old adult C57BL/6 mice as model of cardiac hypertrophy	200 μg/100 μL of Nor-EVs or Hy-EVs were injected into mice through caudal vein once a week, from one week after surgery for 3 weeks			
Human UC-MSC-Exos	Premature ovarian insufficiency	KGN cells as model of POI	Nor-Exos and hy-Exos (50 µg/mL)	- Enhance mitochondrial function and regulate mitochondrial oxidative stress - Improve body weight, ovarian weight coefficient, estrous cycles, ovarian morphology, ovulation count, and sex hormone levels in POI rats	- Increase expression of *SOD2*, *SIRT3*, *PGC1-a*, and *TFAM* - Decrease cell apoptosis by downregulation of caspase-3, caspase-9, BAX, and P53	[101]
		8-week-old SD female rats’ model of POI	200 µL of Hy-Exos or Nor-Exos (1 × 10^9^ cells) was transplanted into each ovary for two weeks (once a week)			
Mouse MSC /MSC-Exo	Cigarette smoking induced mitochondrial dysfunction	lung epithelial cells (BEAS2B cells) exposure to cigarette smoke (CS)	Not specified	- Protective response against the CSE-altered mitochondrial respiration	- Increase the expression of fusion genes (mfn1, mfn2 and opa1) and mitochondrial homeostasis gene (rhot1 gene)	[102]
Mouse HF-MSC-Exos	Ulcerative colitis (with high mitochondrial fission/fusion)	LPS-treated mouse MODE-K cells as a model of ulcerative colitis	Not specified	- Alleviate mitochondrial dysfunction and oxidative stress - Maintain mitochondrial dynamic stability and enhance mitophagy - Ameliorate colonic mucosal damage and inflammatory cell infiltration	- Reduce the expression of HSP60, TOMM20, Drp1 and Fis1 - Increase expression of Mfn1, Mfn2 and OPA1 - increase LC3 expression and colocalization with COX IV - Reduce *IL-1β* and *TNF-α* expression, and increase *IL-4* and *IL-10* expression - miR-214-3p-mediated inhibition of the PI3K/AKT/mTOR signaling pathway	[103]
		4–6 weeks C57BL/6J mice	100 μg of Nor-Exos and Hy-Exos were injected via the tail vein			
Human Ad-MSC-Exos	ALI	MH-S mouse macrophage cells	Exosomes (10 μg/mL)	- Improve macrophages’ mitochondrial integrity and oxidative phosphorylation level - Mitigate lung inflammatory pathology	- Transfer mitochondrial components - Increase mtDNA and MMP	[104]
Human UC-MSC-Exos	IVDD	Human degenerative Nucleus pulposus cells (NPCs)	UCMSC-exos (10^11^ particles/mL)	- Improve viability of NPCs - Improve mitochondrial function - Delay the progression of IVDD in rats	- Reduce ROS and mitochondrial superoxide levels - Increase MMP - Restore the expression of the extracellular matrix proteins, COL2A1 and matrix metalloproteinase-13	[94,95]
		23-month-old male Sprague–Dawley rats as IVDD model (by IVD puncture)	10 uL of UCMSC-Exos (10^11^ particles/mL) were injected into the punctured discs every 2 weeks for 2 months			
Human P-MSC-EVs	CKD	HK-2 as induced model of CKD	Norm-EVs or Hypo-EVs (100 μg/mL)	- Reduce renal fibrosis - Enhance mitochondrial fatty acid oxidation (FAO) - Restore mitochondrial homeostasis	- Reduce collagen I and α-SMA - Restore expression of a FAO key rate-limiting enzyme, carnitine palmitoyl-transferase 1A (CPT1A) - Repair mitochondrial structure, restore mitochondrial mass and ATP production, inhibit oxidative stress, and increase mitochondrial membrane potential	[98]
		Male C57BL/6 SPF mice (7–9 weeks old) were used as a model of ischemia–reperfusion (I/R)-induced renal fibrosis	100 μg of Norm-EVs or Hypo-EVs in 0.15 mL of PBS solvent was administered by tail vein injection immediately after the surgery and on day (D) 1 postsurgery in the Norm-EVs and Hypo-EVs groups, respectively			
Human P-MSC-EVs	Acute kidney injury	TEC line (HK-2) as model of oxidative stress	40–120 μg P-MSC-EVs	- Restore renal function - Restore mitochondrial function (elevate ATP production, reduce mitochondrial ROS and restore mitochondrial mass)	- Modulate inflammation and inhibit apoptosis (downregulation of Kim-1, TNF-α, nf-kb, caspase 8, caspase 9, and bax) - Increase the expression of Nrf2 and SOD2 and decrease the expression of Keap1 - Reduce mitochondrial fragmentation and normalize the mitochondrial potential - Increase mtDNA copy number	[97]
		6–8-week-old male FVB mice as a model of ischemia/reperfusion-induced AKI	0.1 mL of 80 μg of EVs was injected intravenously into AKI mice			

HF-MSC-Exos; hair follicle mesenchymal stem cell-derived exosomes, Hy-Exos; hypoxic exosomes, Nor-Exos; normoxic exosomes, Nor-EVs; normoxic extracellular vesicles, Hy-EVs; hypoxic extracellular vesicles, BM-MSC; bone marrow mesenchymal stem cells, MELAS; mitochondrial encephalomyopathy, lactic acidosis, and stroke-like episode, AdMSC; adipocyte-derived mesenchymal stem cells, ALI; acute lung injury, UC-MSC; umbilical cord mesenchymal stem cells, IVDD; Intervertebral disc degeneration, COL2A1; collagen type II alpha-1, MMP; mitochondrial membrane potential, TECs; tubular epithelial cells, OM-MSCs; olfactory mucosa mesenchymal stem cells, IhOM-MSCs; Ischemic-hypoxic primed OM-MSCs, POI; premature ovarian insufficiency, AKI; acute kidney injury, SOD2; superoxide dismutase 2, HO-1; heme oxygenase 1, P-MSC-EV; placenta-derived MSC-EV, CKD; chronic kidney diseases.

### 5.3. Summary of Experimental Evidence on MSC- and MSC-EV-Mediated Restoration of Mitochondrial Function

Mesenchymal stromal cells from various tissue sources—including bone marrow, umbilical cord, adipose tissue, and olfactory mucosa—have shown strong potential in restoring mitochondrial function across multiple disease models [85,86,87,89,91,95,102,103,104,105]. The primary mechanism underlying these effects involves the transfer of functional mitochondria from MSCs to damaged cells, leading to an increased proportion of healthy versus dysfunctional mitochondria [43,85,105]. These healthy mitochondria shift cellular metabolism from glycolysis toward oxidative phosphorylation, thereby increasing mitochondrial membrane potential, enhancing ATP production, and reducing oxidative stress [43]. MSCs also exert potent antioxidant effects by upregulating enzymes such as SOD2 and HO-1, and they promote anti-apoptotic activity through increased expression of Bcl-2 and decreased expression of pro-apoptotic proteins, including BAX, IL-1β and IL-18 [89]. In addition, MSCs exhibit strong anti-inflammatory effects by suppressing M1 macrophage polarization and downregulating inflammatory mediators such as the NLRP3 inflammasome, ASC, caspase-1, caspase-8 and GSDMD [86,89]. Furthermore, MSCs provide mitochondrial protection by upregulating GRP78, a key regulator of mitochondrial homeostasis that maintains the balance between fusion and fission, prevents excessive Ca^2+^ accumulation, reduces free radical production, preserves mitochondrial membrane potential, and sustains respiratory activity [106,107]. Through these combined mechanisms, MSCs mitigate oxidative injury, suppress inflammation, restore cellular bioenergetics, and promote tissue regeneration in both metabolic and degenerative disorders associated with mitochondrial dysfunction.

Similar to their parent mesenchymal stromal cells, MSC-EVs enhance mitochondrial function by restoring mitochondrial membrane potential and increasing ATP synthesis, while concurrently reducing oxidative damage and apoptosis. MSC-EVs also promote tissue protection through modulation of redox homeostasis, cellular metabolism, and pro-survival signaling pathways. Across various disease models, MSC-EVs have been shown to improve organ function, attenuate tissue fibrosis, and facilitate recovery of normal physiological activity by stabilizing mitochondrial dynamics and preventing progressive damage [94,95,97,98,99,100,101,102,103,104]. Furthermore, mitochondria-enriched extracellular vesicles (ME-EVs) have been developed as a cell-free alternative to MSC transplantation and were shown to exhibit no detectable systemic or retinal toxicity in vivo, whereas MSC transplantation was associated with retinal structural alterations, inflammatory responses, and reduced durability of therapeutic effects, supporting the improved safety profile and stability of EV-based therapy [99]. Collectively, these findings support MSC-EVs as a promising cell-free therapeutic platform that preserves much of the mitochondrial and regenerative efficacy of their parent cells while offering improved safety and stability profiles.

Mechanistically, MSC-EVs mediate their effects through the transfer of bioactive molecules—including proteins and nucleic acids—that stimulate mitochondrial homeostasis and antioxidant defense. In an in vitro model of cardiac hypertrophy, MSC-EVs delivered DJ-1, a cytoprotective protein that defends cardiomyocytes against oxidative stress, maintains mitochondrial integrity and inhibits cell death during ischemia–reperfusion injury (IRI) [108]. Transferred DJ-1 enhanced the expression of antioxidant-related proteins such as NRF2, HO-1, SOD2 and GPX4, while reducing NOX4 expression [100]. Moreover, MSC-EVs increased the expression of SIRT3, TFAM, AMPKα, and PGC-1α, which together form a regulatory network that suppresses oxidative stress and enhances cellular energy production [101]. Similar to their parent cells, MSC-EVs exert anti-apoptotic effects by downregulating caspase-3, caspase-8, caspase-9, BAX, and P53, and exhibit anti-inflammatory actions through the suppression of NF-κB, TNF-α, and IL-1β, along with upregulation of IL-4 and IL-10 [101,102,103]. MSC-EVs also promote mitochondrial fusion by increasing the expression of MFN1, MFN2, and OPA1. Furthermore, under stress conditions, MSC-EVs reduce the expression of HSP60, TOMM20, Drp1 and Fis1, while increasing rhot1 expression to regulate cellular responses to stress and maintain mitochondrial homeostasis [102]. Regulatory miRNAs—such as miR-214-3p, from HF-MSC-exosomes—have been shown to inhibit the PI3K/AKT/mTOR pathway, thereby alleviating mitochondrial dysfunction and oxidative stress. Additional signaling pathways, including the p38 and ERK1/2 pathways, are also modulated by MSC-EV bioactive components.

Emerging evidence indicates that priming and engineering strategies can substantially enhance the functional quality and therapeutic efficacy of MSC-derived extracellular vesicles. Beyond indirect paracrine effects, MSC-EVs have also been shown to mediate direct mitochondrial transfer [99,108]. In a recent study, engineered large mitochondria-enriched extracellular vesicles (Super-EV-Mito) efficiently delivered functional mitochondria and restored mitochondrial bioenergetics in cellular and animal models of mitochondrial disease, highlighting EV size and engineering as critical determinants of effective mitochondrial delivery [99]. Mitochondria-enriched EVs, particularly from iPSC-derived cardiomyocytes, have shown a reliable ability to enhance ATP generation, reduce oxidative stress, and reverse functional impairment in damaged cardiac tissue [109]. Moreover, accumulating evidence indicates that hypoxia-primed MSC-EVs exhibit enhanced mitochondrial rescue compared with EVs derived from normoxic MSCs, suggesting that hypoxic priming enriches bioactive cargo and augments the therapeutic potency of MSC-EVs [98,100,101,103]. Consistently, Vitale et al. demonstrated that hypoxia-conditioned MSCs release functionally active EVs capable of promoting tissue remodeling and supporting repair across multiple injured organs, including the brain, liver, spinal cord, kidney, and heart [110,111]. Collectively, MSC-EVs restore mitochondrial health by integrating antioxidant, anti-apoptotic, anti-inflammatory, and metabolic reprogramming mechanisms, offering an effective therapeutic strategy for conditions driven by mitochondrial dysfunction.

**Figure 2:**
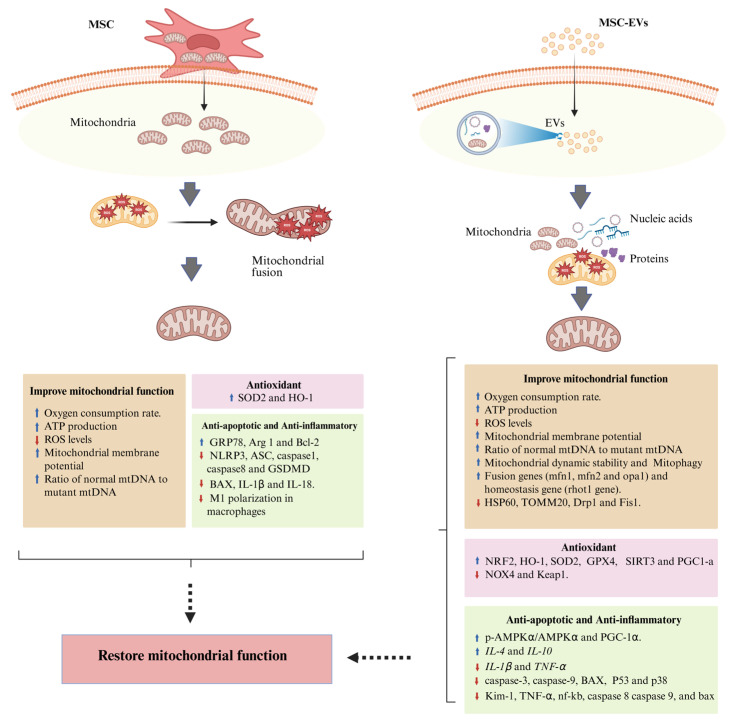
Mechanistic overview of how MSCs and MSC-derived EVs exert therapeutic effects in mitochondrial dysfunctions. MSCs can directly transfer intact, functional mitochondria to defective cells, which can fuse with the recipient’s damaged mitochondria, restore bioenergetics, increase ATP production, improve membrane potential, reduce ROS, and shift the ratio toward healthy mtDNA. MSCs also activate antioxidant, anti-apoptotic, and anti-inflammatory pathways. In contrast, MSC-derived extracellular vesicles primarily exert their effects by delivering a diverse repertoire of bioactive cargo, including mitochondrial proteins, metabolic enzymes, nucleic acids, and regulatory RNAs, and under specific experimental conditions, may also mediate mitochondrial transfer. MSC-EVs modulate mitochondrial quality control by enhancing mitochondrial dynamics and mitophagy, regulating fusion and fission machinery, maintaining mitochondrial homeostasis, and activating robust antioxidant and anti-inflammatory signaling pathways. Collectively, these mechanisms enable MSC-EVs to support mitochondrial repair and functional recovery without direct cellular engraftment. Together, both MSCs and MSC-EVs contribute to mitochondrial restoration, but via distinct therapeutic mechanisms: MSCs provide whole-organelle replacement, whereas EVs mainly provide molecular components and signaling cues that support mitochondrial repair and functional recovery. Created in BioRender. Hasyahril, M. (2026) (https://BioRender.com/4ofqv30). (accessed on 20 November 2025).

Figure 2 illustrates the mechanisms through which MSCs and MSC-EVs may exert their mitochondrial therapeutic effects.

## 6. Challenges and Prospects of MSC- and MSC-EV-Based Therapies for Mitochondrial Dysfunctions

Although growing evidence indicates that MSCs and MSC-EVs can restore mitochondrial function and hold therapeutic promise for mitochondrial disorders, several challenges must be addressed before these approaches can be translated into practical clinical treatments. Current studies demonstrate that both MSCs and their extracellular vesicles contribute significantly to the restoration of mitochondrial function in secondary mitochondrial dysfunction (SMD), primarily through direct mitochondrial transfer or the delivery of mitochondrial components. Because SMDs commonly arise as a consequence of chronic diseases, inflammatory conditions, or aging, the antioxidative, anti-apoptotic, and anti-inflammatory properties of MSCs and MSC-EVs position them as promising candidates for reversing disease progression and improving mitochondrial function and tissue repair [86,87,89,95]. However, data specific to primary mitochondrial diseases remains limited. Existing investigations have largely focused on MSC-based interventions, with very limited studies to date evaluating the therapeutic potential of MSC-EVs in this context, partly because conventional small extracellular vesicles, including exosomes, have limited capacity to encapsulate and deliver intact mitochondria. This gap raises important questions: Can MSC-EVs reproduce the mitochondrial restorative effects observed with MSCs in primary mitochondrial disorders? And do their bioactive cargos have the capacity to enhance antioxidant defenses, ATP production, and mitochondrial biogenesis in genetically impaired cells? Can EV engineering enhance their capacity to carry intact mitochondria?

Furthermore, available data show that MSCs provide a clear functional rescue in mitochondrially impaired cells by delivering healthy mitochondria and wild-type mtDNA, resulting in reduced oxidative stress and mitigation of downstream dysfunction. Although these findings offer strong evidence of metabolic support and restoration of cellular homeostasis, no correction of the underlying mtDNA or nDNA mutations has been observed. The absence of genomic repair indicates that the therapeutic effect is compensatory rather than curative, which may limit the long-term durability of responses in disorders driven by persistent genetic defects. Future therapeutic strategies may integrate MSC- or MSC-EV-based mitochondrial support with targeted gene therapy to correct underlying mtDNA or nDNA mutations. In this integrated approach, MSCs or their EVs would provide rapid metabolic rescue through delivery of healthy mitochondria and bioactive cargos, whereas gene-editing platforms (e.g., CRISPR-based systems or mito-targeted nucleases) would ensure durable correction of the primary defect. Such a combinatorial strategy may overcome the limitations of purely compensatory therapies and advance MSC/MSC-EV applications from transient support toward truly disease-modifying interventions in mitochondrial disorders.

A growing body of evidence indicates that mesenchymal stromal cells can restore mitochondrial function by transferring healthy mitochondria to damaged cells [85,105]. However, the precise mechanisms underlying these therapeutic effects remain incompletely understood and require further exploration. Critical gaps include whether the transferred mitochondria act independently to supply ATP and counter oxidative stress, whether they fuse with endogenous dysfunctional mitochondria to restore network integrity, or whether they stimulate mitophagy to clear damaged organelles. It also remains unknown whether these donated mitochondria can replicate and sustain long-term function, a process that requires coordinated mtDNA replication and nuclear-encoded protein import. The durability of their effects within defective cells likewise remains an open question. In parallel, MSC-derived EVs have shown the ability to improve mitochondrial function, yet the mechanisms responsible for these effects are still in need of exploration and discussion. Key questions include whether MSC-derived extracellular vesicles can effectively transfer intact mitochondria under physiological conditions or whether this process requires induction through priming or bioengineering strategies, and how priming conditions or MSC source influence mitochondrial cargo composition and functionality. Together, these unresolved issues highlight several priority areas for immediate future investigation, including the fate and functional integration of transferred mitochondria, the molecular composition of MSC-EV mitochondrial cargo, and the temporal stability of these therapeutic effects in impaired cells.

Beyond mechanistic uncertainties, manufacturing constraints—particularly those related to cell production and scalability—also limit clinical translation. MSC expansion is limited by finite passage numbers, donor variability, senescence, and increasing batch heterogeneity, all of which restrict large-scale manufacturing and complicate regulatory compliance. To address these challenges in MSC production, recent studies have introduced a pluripotent stem cell-based method for MSC induction. Human pluripotent stem cell-derived MSCs exhibit superior expansion capacity (exceeding 30 passages), enhanced proliferative potential, resistance to senescence, and robust secretory profiles, including increased cytokine and exosome production [112]. Moreover, the inherent heterogeneity of MSC populations complicates standard culture practices, making them inefficient and resource-intensive. To overcome this limitation, recent advances have led to the development of high-purity MSCs known as rapidly expanding clones (RECs), derived from single MSCs. These RECs demonstrate improved homogeneity, superior mitochondrial integrity, and a stronger capacity to restore bioenergetics and mitochondrial function in deficient cells [113]. In contrast, EVs can be produced from standardized MSC sources with high scale, greater batch-to-batch consistency and reduced biological variability [65,69]. Moreover, EVs demonstrate superior stability compared with MSCs; they tolerate multiple freeze–thaw cycles, maintain bioactivity during long-term storage, and can be formulated as off-the-shelf medicinal products without the viability constraints or complex cryopreservation requirements associated with living cells [112,113]. These attributes significantly reduce production costs, simplify storage and transport logistics, and facilitate the development of standardized, scalable EV-based therapies.

Advances in production must be accompanied by rigorous evaluation of safety. Although several studies suggest that MSC- and MSC-EV-based therapies are generally well tolerated, their long-term safety profile remains unclear. Existing clinical and preclinical reports indicate minimal immune reactivity and a low risk of tumorigenicity [114]. For example, Vega-Letter et al. demonstrated that xenogeneic mitochondrial transplantation did not elicit adaptive immune responses and even exerted anti-inflammatory and tissue-protective effects in osteoarthritis models [115]. Similarly, the Stem Cell Ophthalmology Treatment Study (SCOTS), which evaluated MSC therapy for mitochondrial optic neuropathies, reported no adverse events during a 12-month follow-up period [116,117,118,119]. However, such short observation windows cannot exclude delayed adverse effects, genomic instability, or long-term immunological consequences. In this context, MSC-derived extracellular vesicles may offer a comparatively safer alternative to whole-cell therapy. MSC-EVs exhibit markedly lower immunogenicity and negligible tumorigenic potential while preserving many of the therapeutic functions of their parent cells. They can suppress T- and B-cell proliferation, modulate macrophage activity, inhibit NK-cell responses, and reduce immune rejection [120,121]. Nevertheless, despite these advantages, comprehensive long-term safety assessments remain lacking for both MSCs and MSC-EVs, underscoring the need for extended follow-up studies and standardized safety monitoring in future clinical applications.

Another obstacle for clinical translation of MSC- and MSC-EV-based therapies is the complexity of primary mitochondrial disorders, which often involve multisystem dysfunction. Although MSCs have demonstrated the ability to home to distant organs such as the brain, heart, lungs, and kidneys [122], it remains uncertain whether this capacity is sufficient for diseases that affect multiple organs simultaneously. This may raise pivotal questions: Will the homing efficiency of MSCs and MSC-EVs be adequate in multi-organ contexts? Can the same dosing strategies, preparation methods, and routes of administration used in single-organ involvement in SMDs be applied to PMDs? Addressing these questions will require robust in vivo disease models to determine optimal dosage, delivery routes, and treatment duration, as well as to evaluate therapeutic efficacy and biodistribution.

Taken together, the therapeutic application of MSCs and MSC-EVs in mitochondrial dysfunctions remains an active and critically important area of investigation. Emerging technologies in cell-free regenerative medicine—such as bioengineered EVs and hypoxia priming—are expected to enhance therapeutic efficiency, safety, and scalability. Future directions should focus on:

- Defining the molecular mechanisms of mitochondrial repair mediated by MSCs and EVs.
- Developing standardized protocols for MSC/EV production, storage, and administration.
- Establishing multi-organ disease models to evaluate homing capacity, dosing requirements, and biodistribution.
- Conducting long-term safety and efficacy studies to assess immune tolerance and functional recovery.

With continued refinement and validation, MSCs and their EVs may provide a viable platform for developing targeted therapies for mitochondrial diseases, helping to narrow the gap between current symptomatic management and future mechanism-based treatments.

## 7. Conclusions

Mitochondrial dysfunction represents a major barrier to cellular homeostasis and contributes to a wide range of degenerative, inflammatory, and genetic disorders. Growing evidence demonstrates that mesenchymal stromal cells and their extracellular vesicles can restore mitochondrial function through mechanisms that include mitochondrial transfer, delivery of mitochondrial components, and modulation of oxidative stress and inflammatory signaling. Although these findings highlight the therapeutic promise of MSC- and MSC-EV-based interventions, the precise mechanisms governing their actions, durability, and integration within defective cells remain incompletely understood. Furthermore, despite encouraging short-term safety data, long-term safety and immunological consequences of both approaches require systematic investigation. MSC-EVs—with their lower immunogenicity, greater stability, and scalable production—represent a compelling cell-free alternative to MSC therapy; however, their mitochondrial cargos and mechanistic pathways remain to be fully elucidated. Overall, MSCs and MSC-EVs hold significant potential as next-generation therapeutics for mitochondrial disorders, but translating this promise into clinically effective, durable, and safe treatments will require closing existing knowledge gaps and advancing both mechanistic understanding and regulatory-ready production platforms.

## Figures and Tables

**Table 1:** Key genetic basis, pathophysiological mechanisms, and key clinical features of examples of primary and secondary mitochondrial dysfunctions.

MD Class	Disease/Syndrome	Key Genetic Basis	Core Pathophysiological Mechanisms	Key Clinical Features	Refs.
PMD	Leigh syndrome	Mutation in mtDNA genes (e.g., MT-ND1, MT-ND3, MT-ND4, and MT-ND6) and/or nDNA genes (e.g., NDUFS1)	Impaired respiratory chain complexes synthesis (often complex I), impaired ATP, lactic acidosis, neurodegeneration	Infant/child onset; developmental regression, seizures, brainstem/basal ganglia signs	[4,42]
	MELAS	mtDNA mutation in MT-TL1 gene	Impaired respiratory chain complexes synthesis, particularly complexes I and IV, impaired ATP, oxidative stress and reduced mitophagy	Stroke-like episodes (paralysis, vision loss, cortical blindness or deafness), migraine-like headaches, vomiting, seizures, myopathy, fatigue, and psychopathology (depression, psychosis, anxiety, and cognitive decline)	[43]
	LHON	MT-ND1 m.3460G>A; MT-ND4 m.11778G>A; MT-ND6 m.14484T>C	Complex I dysfunction, OXPHOS impairment, impaired ATP, increased mtROS, retinal ganglion cell (RGC) degeneration	Subacute painless central vision loss; bilateral involvement; optic neuropathy	[44]
	NARP	mtDNA mutation in MT-ATP6 gene (m.8993 T>G or m.8993 T>C)	Impaired proton translocation mechanism of ATP synthase (complex V) and subsequent disturbance of OXPHOS	Muscle weakness, sensory neuropathy, ataxia, seizures, dementia, retinitis pigmentosa, optic atrophy, and developmental delay	[45]
	Single large-scale mtDNA deletion syndromes (KSS and Pearson syndrome)	Sporadic single large-scale mtDNA deletions (e.g., 4977 bp deletion)	Impaired respiratory chain complexes synthesis (complexes I, III, IV) as well as multiple mt-tRNAs, OXPHOS impairment, increased oxidative stress	KSS: ophthalmoplegia/ptosis, retinopathy and heart block; Pearson syndrome: sideroblastic anemia, intracerebral bleeding, pancreatic exocrine insufficiency, lactic acidosis, and congenital malformations	[46,47]
	PEO	Single or multiple large-scale mtDNA deletion	OXPHOS disturbance	Adult-onset, progressive bilateral ptosis and diffuse, symmetric ophthalmoparesis	[48]
SMD	Friedreich ataxia	FXN gene defect	Impaired mitochondrial iron metabolism, secondary respiratory chain dysfunction and oxidative stress	Progressive ataxia, neuropathy, cardiomyopathy common	[35]
	Charcot-Marie-Tooth disease type 2A (axonal peripheral neuropathy)	nDNA mutation in MFN2 gene	Impaired mitochondrial fusion, transport, and mitophagy	early childhood onset, peripheral neuropathy; variable optic/CNS involvement	[49]
	Secondary PEO	mtDNA depletion or deletions secondary to mutations in nDNA genes, responsible for mtDNA maintenance, including POLG, POLG2, SLC25A4, C10orf2, SPG7, DNA2, RNASEH1, TOP3A, TK2, DGUOK, RRM2B, GMPR, LIG3, and RRM1	Secondary OXPHOS defects	Progressive bilateral ptosis and diffuse, symmetric ophthalmoparesis	[48]
	Dominant optic atrophy (DOA)	Mutations in *OPA1* gene	Mitochondrial fragmentation, impaired OXPHOS, reduced ATP, and increased ROS, resulting in RGC apoptosis	Childhood-onset, progressive bilateral vision loss and color vision deficits	[50]
	Parkinson disease	PINK1, Parkin, *LRRK2* and *SNCA*	Disrupted mitochondrial fusion/fission balance and impaired mitochondrial mitophagy and quality control lead to mitochondrial fragmentation, accumulation of damaged mitochondria, impaired OXPHOS and neurodegeneration	Early-onset parkinsonism, resting tremors, bradykinesia, rigidity, and postural instability	[33,34]
	Chronic diseases such as DM, CVD, cancer, and neurodegenerative disorders and aging-related diseases such as Alzheimer’s disease (AD)	Polygenic and /or environment (no single-gene)	Increased oxidative stress, abnormal mitochondrial dynamics, impaired biogenesis, and autophagy defects	Disease-related clinical features	[51]

**Table 2:** Therapeutic strategies for mitochondrial diseases.

Therapy/Drug	Mechanism of Action	Clinical Development Stage/Status	Refs.
Coenzyme Q10 (CoQ10)	Electron carrier in ETC; antioxidant; improves ATP production	Widely used supportive therapy; limited RCT evidence	[5,61,62,63,64,65]
Idebenone	Synthetic CoQ10 analog; bypasses complex I defects	Approved in EU for LHON; Phase III trials completed	[64]
Riboflavin (Vitamin B2)	Cofactor for complex I/II flavoproteins	Supportive clinical therapy	[65]
Thiamine (Vitamin B1)	Cofactor for pyruvate dehydrogenase	Supportive clinical therapy	[66]
Elamipretide	Cardiolipin binding; stabilizes inner mitochondrial membrane	Phase III trials in mitochondrial diseases	[67]
Arginine/Citrulline	Enhances nitric oxide; reduces MELAS stroke-like episodes	Supportive clinical therapy	[68]
L-Carnitine	Facilitates fatty acid transport into mitochondria	Supportive clinical therapy	[69]
EPI-743	Synthetic analog of vitamin E; reduce ROS production by affecting redox state of intracellular glutathione	Phase I–II clinical trials	[70]
Sonlicromanol (KH176)	Redox modulator targeting thioredoxin system	Phase I–II clinical trials	[71]
AAV-ND4 Gene Therapy (LHON)	AAV-mediated nuclear expression of ND4 gene	Phase I–III clinical trials	[71,72,73]
mtDNA Editing (mtZFNs, mitoARCUS nuclease)	Selective elimination/correction of mutant mtDNA	Preclinical research stage	[74,75]

## Data Availability

No new data were created or analyzed in this study. Data sharing is not applicable to this article.
